# Epigenetic Changes in Basal Cell Carcinoma Affect SHH and WNT Signaling Components

**DOI:** 10.1371/journal.pone.0051710

**Published:** 2012-12-17

**Authors:** Tjinta Brinkhuizen, Karin van den Hurk, Véronique J. L. Winnepenninckx, Joep P. de Hoon, Ariënne M. van Marion, Jürgen Veeck, Manon van Engeland, Maurice A. M. van Steensel

**Affiliations:** 1 Department of Dermatology, Maastricht University Medical Center, Maastricht, The Netherlands; 2 Department of Pathology, Maastricht University Medical Center, Maastricht, The Netherlands; 3 Department of Clinical Genetics, Maastricht University Medical Center, Maastricht, The Netherlands; 4 GROW-School for Oncology and Developmental Biology, Maastricht University, Maastricht, The Netherlands; 5 Department of Internal Medicine (Division of Medical Oncology), Maastricht University Medical Center, Maastricht, The Netherlands; 6 Department of Pathology, VieCuri Medical Centre, Venlo, The Netherlands; 7 Institute of Pathology, University Hospital of the RWTH Aachen, Aachen, Germany; University of Navarra, Spain

## Abstract

**Background:**

The genetic background of Basal Cell Carcinoma (BCC) has been studied extensively, while its epigenetic makeup has received comparatively little attention. Epigenetic alterations such as promoter hypermethylation silence tumor suppressor genes (TSG) in several malignancies.

**Objective:**

We sought to analyze the promoter methylation status of ten putative (tumor suppressor) genes that are associated with Sonic Hedgehog (SHH), WNT signaling and (hair follicle) tumors in a large series of 112 BCC and 124 healthy control samples by methylation-specific PCR.

**Results:**

Gene promoters of *SHH* (P = 0.016), adenomatous polyposis coli (*APC*) (P = 0.003), secreted frizzled-related protein 5 (*SFRP5*) (P = 0.004) and Ras association domain family 1A (*RASSF1A*) (P = 0.023) showed significantly more methylation in BCC versus normal skin. mRNA levels of these four genes were reduced for *APC* and *SFRP5* in BCC (n = 6) vs normal skin (n = 6). Down regulation of SHH, APC and RASSF1A could be confirmed on protein level as well (P<0.001 for all genes) by immunohistochemical staining. Increased canonical WNT activity was visualized by β-catenin staining, showing nuclear β-catenin in only 28/101 (27.7%) of BCC. Absence of nuclear β-catenin in some samples may be due to high levels of membranous E-cadherin (in 94.1% of the samples).

**Conclusions:**

We provide evidence that promoter hypermethylation of key players within the SHH and WNT pathways is frequent in BCC, consistent with their known constitutive activation in BCC. Epigenetic gene silencing putatively contributes to BCC tumorigenesis, indicating new venues for treatment.

## Introduction

Basal cell carcinoma (BCC) accounts for 75% of all skin cancers and is the most frequent malignancy in Caucasians. Its incidence is rising by 3–8% each year [1], resulting in an average lifetime risk for Caucasians of developing BCC of 30% [2], [3]. BCC rarely metastasize [4], but when left untreated they may cause extensive local tissue destruction [4], [5]. Surgical excision is the current standard treatment, with average costs in the Netherlands of €900 per procedure, amounting to a total of €45 million by 2015 [6], [7]. The inevitably rising workload can be expected to stress the health care system even further. Hence, BCC is becoming a serious health problem. There is a clear need for a simple and cost-efficient medical treatment. In order to develop one, a thorough understanding of BCC pathobiology will be required and the past few years have witnessed considerable progress in this respect.

Inappropriate activity of the Sonic Hedgehog (SHH) pathway due to mutations of its component genes is firmly implicated in BCC pathogenesis. Inactivating mutations of Patched-1 (*PTCH1)* are the most common (30–60%) in sporadic BCC [8]–[11], followed by mutations (10–20%) in Smoothened (*SMO*) or Suppressor of Fused (*SU(FU)*) [11]–[16]. Even though mutations of the SHH target gene, Glioma-associated oncogene homolog-1 (*GLI1)*, are uncommon, approximately 90% of sporadic BCC overexpress *GLI1,* which contributes to tumor growth [17]. In addition, Yang and colleagues proved the wingless-type MMTV integration site family (WNT) pathway to be essential in tumorigenic response to deregulated SHH signaling, suggesting crosstalk between the SHH and canonical WNT pathways in BCC [18]. Non-canonical WNT signaling, however, might also be active in the context of repressed canonical WNT signaling in BCC, as recently reported by Pourreyron et al. [19]. Thus, both WNT signaling pathways might act in a mutually antagonistic fashion in driving BCC growth. In all, the genetic aspects of BCC development seem to be well defined. However, most if not all cancers are characterized by epigenetic alterations in addition to genetic changes [20]. Epigenetic modifications are heritable changes in deoxyribonucleic acid (DNA) structure other than alterations in the DNA sequence, and when pathologically altered are capable of driving malignant tumor development and progression. To date, DNA hypermethylation is the best-characterized epigenetic mechanism, comprising the addition of a methyl group to a cytosine base adjacent to a guanine base (the CG dinucleotide) [20]. CG-rich areas (CpG islands) located in the promoter region of many genes become hypermethylated in numerous malignancies [21] leading to gene silencing [22]. Promoter hypermethylation can precede genetic mutations and genomic instability in tumor development, and may thus not only be crucial for carcinogenesis, but also represent a potential therapeutic target [23]. Indeed, DNA demethylating agents, such as 5-azacytidine (Vidaza®, Celgene) and 5-aza-2′-deoxycytidine (Dacogen™, MGI Pharma) can lead to reactivation of silenced genes [24]. Thus, it would be of interest to determine the contribution of promoter hypermethylation to BCC pathogenesis. To date, a very restricted number of studies have addressed this question in a limited number of samples [25]–[30]. Therefore, we decided to assess the promoter CpG island methylation status of nine tumor suppressor genes (TSGs). Patched Homolog 1 (*PTCH1)* is the SHH receptor and is the most commonly mutated tumor suppressor in BCC, whereas adenomatous polyposis coli *(APC)*, secreted frizzled-related protein 1 *(SFRP1)*, *SFRP2*, *SFRP4,* and *SFRP5* are all negative regulators of the canonical WNT pathway. Sustained signaling through the canonical WNT pathway contributes to the development of colorectal cancer as well as basal cell carcinoma [18], [19], [31]. Since BCC are considered as hair follicle tumors, we additionally analyzed the cylindromatosis (*CYLD)* and tuberous sclerosis-1 (*TSC1)* genes, which both are TSGs known to be involved in classic hair follicle tumor syndromes [32], [33]. Additionally, TSC complex proteins are crucial negative regulators of mTOR activity, which is implicated in tumor growth [34]. Ras association domain family 1A *(RASSF1A)* is a well-known TSG, promoter methylation of which has been described as an early and frequent event in several malignancies including basal cell carcinoma [28], [35]. Finally we examined *SHH*, since we hypothesized that downstream activation of its signaling pathway would permit it to become inactivated through methylation.

We have demonstrated significant hypermethylation affecting several of the selected genes in BCC, supporting the involvement of epigenetic aberrations in the most common skin cancer.

## Materials and Methods

### Primary Tissue Specimens

The methylation status of ten selected genes was examined in 112 sporadic BCC samples (107 formalin-fixed paraffin-embedded (FFPE), five fresh frozen (FF)) diagnosed at the Department of Dermatology, MUMC+. All BCC samples were obtained from the Maastricht Pathology Tissue Collection (MPTC). Distinct histological subtypes included were: superficial (sBCC) (*n* = 37), nodular (nBCC) (*n = *53), and infiltrative (iBCC) (*n = *22). Thirty of these samples were used for immunohistochemical analysis of the expression of SHH, APC and RASSF1A. β-catenin and E-cadherin were assessed on respectively 101 (30 sBCC, 40 nBCC, 31 iBCC) and 59 BCC (17 sBCC, 27 nBCC, 15 iBCC) independent samples. Patients included 58 men (mean age 68.1 years, SD ±11.0) and 54 women (mean age 66.2 years, SD ±15.1) A total of 124 healthy control tissue samples (71 FFPE, 53 FF) was collected at autopsies and matched with the 112 BCC patients with regard to age and gender (Table 1). Relatives gave their written consent for usage of the skin tissues. For 31 of these controls, no data were available concerning age and gender. Collection, storage and use of all tissues and patient data were performed in agreement with the “Code for Proper Secondary Use of Human Tissue in the Netherlands”. All of the used samples and corresponding data were de-linked and anonymized. Usage of both BCC and control tissue samples was approved by the MPTCscientific committee (MPTC 2009-05).

**Table 1:** Sample characteristics.

	Tumor (n = 112)	Normal skin (n = 124)
**Sex**		
** Male, n (%)**	58 (51.3)	50 (53.8)
** Female. n (%)**	54 (48.2)	43 (46.2)
** Unknown, n (%)**	0 (0)	31 (25.0)
**Age, years mean**		
** Overall**	67.2 (±13.1 SD)	68.1 (±14.0 SD)
** Man**	68.1 (±11.0 SD)	67.9 (±12.2 SD)
** Women**	66.2 (±15.1 SD)	68.4 (±16.0 SD)
**Tissue Source**		
** FFPE, n (%)**	107 (95.5)	71 (57.3)
** FF, n (%)**	5 (4.5)	53 (42.7)
**BCC subtypes, n (%)**		
** Superficial**	37 (32.7%)	
** Nodular**	53 (46.9%)	
** Infiltrative**	22 (19.6%)	

* FFPE =  Formalin-fixed, paraffin-embedded, FF =  Fresh- frozen.

### DNA Isolation

From each paraffin tissue block and FF sample, a 4 µm section was cut and stained with haematoxylin & eosin (H&E) as a part of the routine intake procedure. All H&E sections were reviewed by a dermato-pathologist (AM or VW) to confirm diagnosis and histological subtype. After deparaffinization of five sections (20 µm) of each FFPE sample, tumor tissue was macroscopically scraped and collected in 1.5 ml tubes. The sections of the FF samples were directly collected into 1.5 ml tubes. Genomic DNA was extracted from FFPE tissue sections by using a Qiagen microkit for DNA isolation (Qiagen, Venlo, The Netherlands) following the manufacturer’s directions.

### Bisulfite Modification of Genomic DNA, Methylation-specific PCR and Bisulfite Genomic Sequencing

Promoter CpG island methylation was determined by bisulfite genomic sequencing (BGS) for *SHH,* and by MSP for *PTCH1*, *SHH*, *APC*, *SFRP1*, *SFRP2¸ SFRP4*, *SFRP5, CYLD, TSC1,* and *RASSF1A.* Sodium bisulfite modification was performed on 500 ng of genomic DNA isolated from the tissue sections by use of an EZ DNA methylation kit (Zymo Research Co, Orange, CA, USA) according to the manufacturer’s instructions. The modified DNA was eluted to 50 ng/µl in H_2_0 and stored at −80°C.

Nested methylation specific polymerase chain reaction (MSP) was performed on bisulfite-modified genomic DNA with primers specific for methylated DNA and unmethylated DNA as previously described [36]–[38]. Nested MSP is exquisitely suitable for methylation analysis of FFPE DNA since it is highly sensitive. Primer sequences and PCR conditions are listed in Table S1. To assess reproducibility of the nested MSP approach, MSP reactions were performed in duplicate starting from DNA amplification with flanking polymerase chain reaction (PCR) primers. Non-concordant MSP results were analyzed a third time and concordance in two out of three assays was accepted as end result. The overall reproducibility for the MSP data was 90.3%.

For BGS analysis, one µl of PCR product from bisulfite-modified genomic DNA was cloned into TOP10 bacteria using a TOPO-TA cloning kit (Invitrogen, Breda, The Netherlands). Bacteria were cultured at 37°C overnight. DNA was extracted from at least ten independent bacterial clones and sequenced using an automated DNA sequencer (Applied Biosystems, Foster City, CA, USA). Sequencing data was analyzed using Sequence Scanner v1.0 software (Applied Biosystems). Mean values were calculated and represented in pie-chart figures per CpG.

### RNA Isolation, Reverse Transcription and Quantitative Reverse Transcription PCR (RT-PCR)

Total ribonucleic acid (RNA) from FF samples containing more than 75% BCC cells (n = 6) as evaluated from H&E stained sections and FF normal skin samples (n = 6) was isolated using the standard procedure for TRIzol® RNA extraction (Invitrogen) and stored at −80°C. Complementary DNA (cDNA) synthesis was performed using the iScript™ cDNA Synthesis kit (Biorad, Veenendaal, The Netherlands) according to the manufacturer’s instructions. Quantitative RT-PCR on 30 ng cDNA was carried out using primer sets for *APC*, *RASSF1A*, *SFRP5*, and *SHH.* Primer sequences and PCR conditions are listed in Table S1. Messenger RNA (mRNA) for *SHH* and *RASSF1A* could not be detected. APC and SFRP5 expression levels for each BCC sample were normalized to the housekeeping gene *Cyclophylin A* and average expression levels in normal skin tissues (n = 6) by 2^−ΔΔCt^ parameter [39]. To assure accuracy, all reactions were performed in triplicate.

### Immunohistochemistry

For SHH, APC and RASSF1A analysis, FFPE sections (4 µm) were deparaffinized in xylene, rehydrated and incubated in 0.3% hydrogen peroxide (H_2_O_2_) in methanol for 30 minutes to inactivate endogenous peroxidase activity. Antigen retrieval was performed by microwave treatment at 90 W for 10 minutes in 10 mM citrate buffer (pH 6) (APC and SHH) or Envision Flex target retrieval solution high pH (Dako, Heverlee, Belgium) (RASSF1A). Next, non-specific protein binding was blocked using 3% bovine-serum-albumin (BSA). Subsequently, the sections were incubated for 1 h at room temperature with primary antibodies listed in table S2. A horseradish peroxidase (HRP)-conjugated second antibody, either PowerVision+ (ImmunoVision Technology, Brisbane, CA, USA) (APC and SHH) or Envision detection system (Dako) (RASSF1A) was applied for 30 minutes. Bound antibody was visualized by using 3,3-diaminobenzidine (DAB) for 10 minutes. Tissue was counterstained with haematoxylin, dehydrated and sealed with coverslips. Phosphate-buffered saline (PBS) was used throughout for washing steps.

For β-catenin and E-cadherin analysis, sections were pre-treated in a pre-treatment module using EnVision FLEX Target Retrieval Solution, High pH (Dako). Staining was performed on a Dako autostainer system using the Dako Envision Flex kit (K8002) for secondary detection. Sections were counterstained with Gill II haematoxylin, dehydrated and sealed with coverslips. For all antibodies, tissue known to be strongly expressing respective protein was included as positive control (Table S2). Negative controls (omission of the primary antibody) were included in all experiments.

### Interpretation of Staining

A specialized dermato-pathologist of the Department of Pathology, MUMC+ (VW) and an experienced resident of the Department of Dermatology, MUMC+ (TB), examined sections independently. Any discrepancy between the observers was discussed and resolved by consensus. β-catenin staining was assessed with respect to localization (membranous, cytoplasmic, and nuclear). For the other antibodies, the percentage positive tumor cells were determined by assessing ten randomly chosen high-power-fields (magnification 200×) per slide and the average of both observers’ values was used for analysis. Additionally, the intensity of E-cadherin was considered with respect to an internal positive control ranging from 0 (no staining) to 1 (weak), 2 (moderate), and 3 (strong) staining.

### Statistics

Statistical analyses were carried out using SPSS version 18.0 software (SPSS, Chicago, IL, USA). Discrete data were analyzed using a Chi-square or Fisher’s exact test, where the independent-samples T-test was used for continuous variables. The correlation between two discrete variables was evaluated by the Cohen’s kappa [40]. To evaluate the effect of methylation on the probability of the presence of tumor, multivariate binary logistic regression analyses were performed with presence or absence of tumor as dependent variable. The two way random effect model with absolute agreement intra-class correlation coefficient (ICC) was used as inter-rater reliability analysis to determine consistency among raters [41]. An ICC ≥0.75 indicates excellent reproducibility [42]. All reported *P* values are two-sided, and *P* values ≤0.05 were considered statistically significant.

## Results

### Methylation of SHH and WNT Pathway Components in BCC

**Figure 1:**
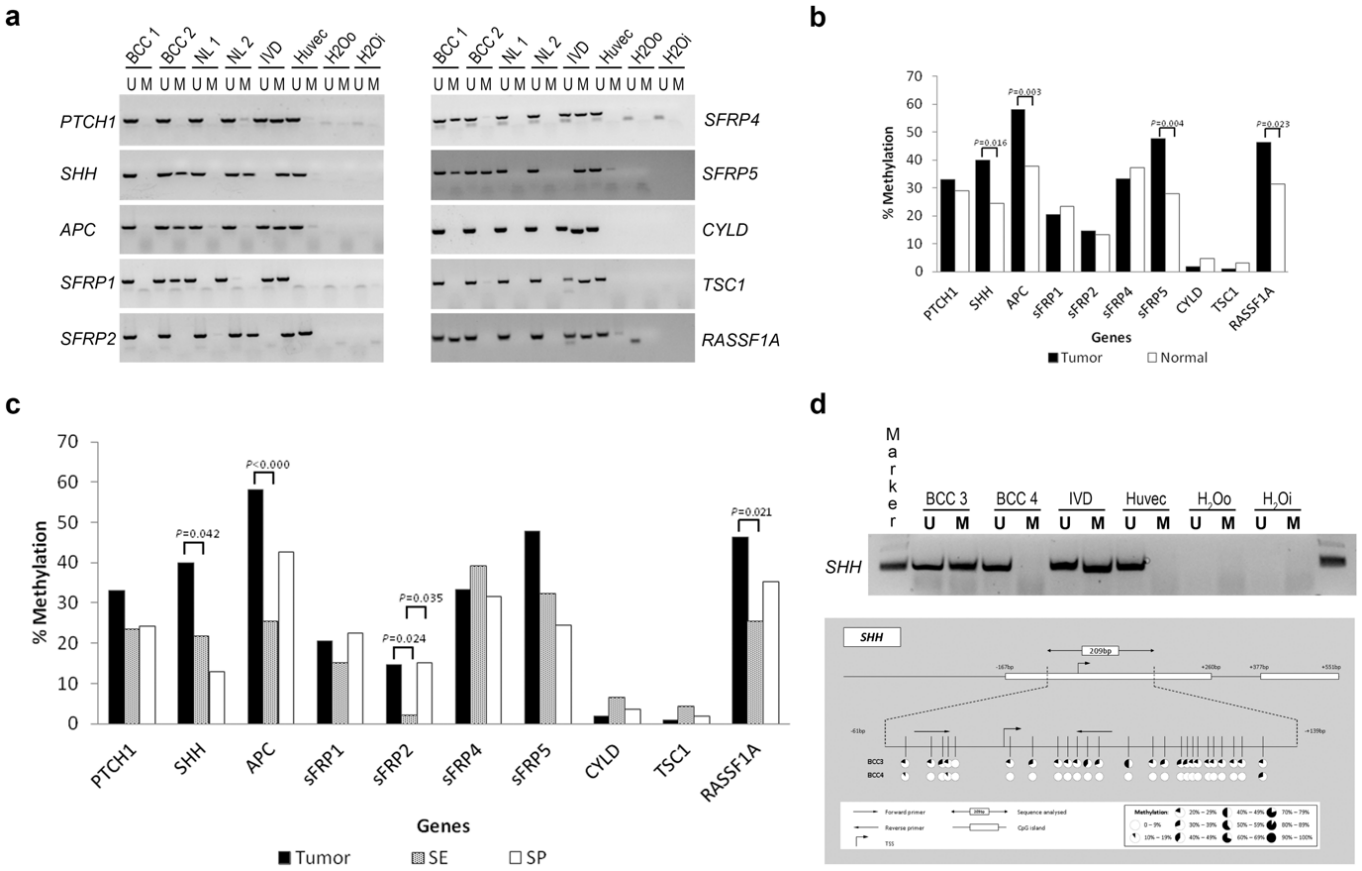
Methylation analysis in BCC and normal skin of ten candidate genes. **A.** Illustration of MSP results of ten candidate genes resolved on a 2% agarose gel**.** BCC: Basal cell carcinoma; NL, normal; IVD, In Vitro Methylated-DNA; Huvec, Human Umbilical Vein Endothelial Cells; U, unmethylated; M, methylated; H_2_0o, water control outside PCR; H_2_0i, water control inside PCR. **B.** Illustration of methylation frequencies of ten candidate genes in BCC (n = 112) and normal skin samples (n = 124). *P*-values represent the difference between percentage methylation in BCC an normal skin. **C.** Illustration of methylation frequencies of ten candidate genes in BCC, sun-exposed (SE) normal skin and sun-protected (SP) normal skin. *P*-values represent both the difference between percentage methylation in BCC and SE and SP. **D.** *SHH* sequence data of bisulfite treated genomic DNA from patients. Upper part shows the *SHH* promoter region starting 1000 base pairs (bp) upstream of the transcription start site (TSS) to 1000 bp downstream. White boxes indicate putative CpG islands (EMBOSS, http://emboss.sourceforge.net). The 256 bp region sequenced stretches from −51 bp from the TSS to +409 bp. Indicated with arrows are the forward and reverse methylation specific PCR (MSP) primers. Vertical bars represent CpG dinucleotides and pie -charts represent the percentage of methylated CpG sites (percentage over at leased 10 sequenced clones).

A total of nine candidate TSGs and one oncogene was examined in a series of primary BCC (n = 112) of three different subtypes and normal skin (n = 124). Characteristics of the tumor and normal skin samples are listed in Table 1. Primers located in promoter CpG islands were previously determined [36]–[38]. Our data showed that promoters of four genes were significantly more frequently methylated in BCC tissue versus normal skin: *SHH* (44/100 (40.0%) vs. 30/122 (24.6%), *P* = 0.016), *APC* (64/110 (58.2%) vs. 47/124 (37.9%), *P* = 0.003), *SFRP5* (52/109 (47.7%) vs. 28/100 (28.0%), *P* = 0.004), and *RASSF1A* (52/112 (46.4%) vs. 39/124 (31.3%), *P* = 0.023. The correlation between methylation in *SHH* and *SFRP5* was moderate (kappa 0.68 (95% CI 0.50–0.78)); no further correlations between genes could be detected. After mutual correction of the two genes *SHH* and *SFRP5* by multivariate logistic regression, odds ratios for individual genes were still larger than 1 (O.R. 1.42 (*P* = 0.304) and 2.01 (*P = *0.032) respectively), suggesting that methylation of *SFRP5* is significantly associated with the occurrence of BCC. Notably, of the four significant differentially methylated genes, nBCC harbored more frequent methylation for *APC* and *RASSF1A*, whereas *SHH* and *SFRP5* were more frequently methylated in iBCC. Nevertheless, none of these differences were significant because of the low sample numbers. Moreover, although *SFRP4* overall was not significantly hypermethylated in BCC, its methylation frequencies did vary according to the subtype (*P = *0.010 for sBCC vs nBCC plus iBCC, *P = *0.011 sBCC vs nBCC) (Figure S1). No significant differential methylation could be detected for *PTCH1*, *SFRP1*, *SFRP2*, *SFRP4*, *CYLD,* and *TSC1* (Figure 1A and 1B). When we compared sun-exposed (SE) (*n* = 47) with sun-protected (SP) (*n* = 54) normal skin tissue, only methylation of *SFRP2* was found to be significantly different, with more frequent methylation in SP skin (SE 2.2% vs. SP 15.1%, *P* = 0.035) (Figure 1C). Gender or age-associated methylation patterns were not identified.

As MSP primers of *APC*, *SFRP5*, and *RASSF1A* had been previously established and validated [38], [43], [44], we only validated our MSP results of the fourth differentially methylated gene, *SHH*, by BGS, which is considered the gold standard [45]. Figure 1D depicts the BGS results of one methylated and one unmethylated *SHH* sample as measured by MSP, and BGS could confirm MSP results.

### Expression of SHH, APC, SFRP5, and RASSF1A is Reduced in BCC

**Figure 2:**
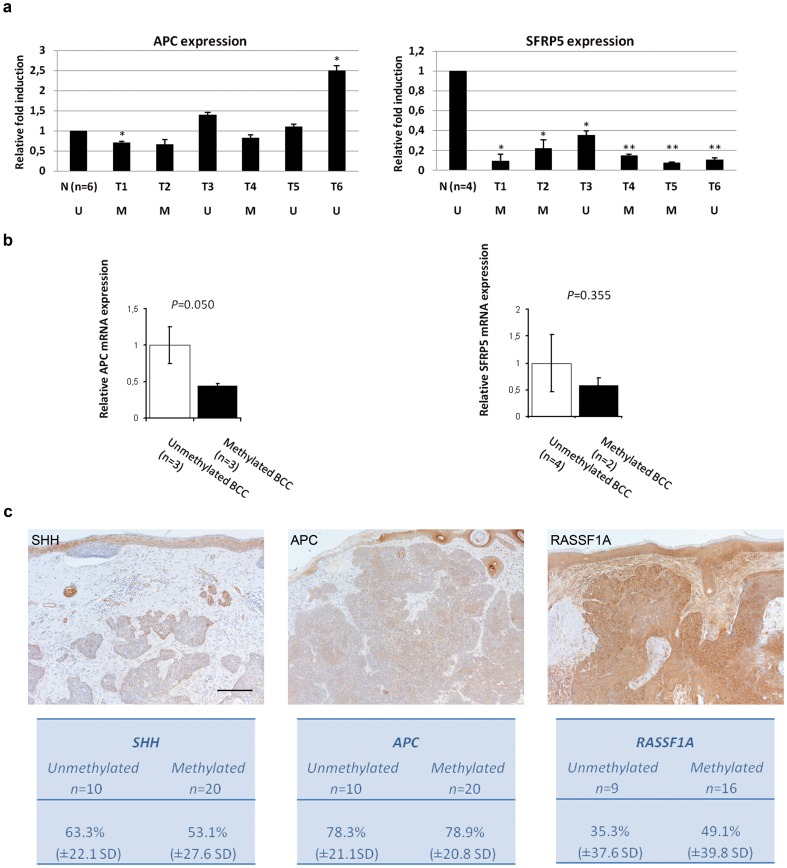
Expression of SHH, APC, SFRP5 and RASSF1A is reduced in BCC versus healthy skin control tissue. **A.** Relative expression levels of *APC* and *SFRP5 i*n BCC tissues as compared to the expression level in normal skin (n = 6) (2−^ΔΔCt^). All reactions were done in triplicates, standard error of the mean (SEM) is shown as error bars. Expression levels were normalized to Cyclophilin A. U, unmethylated sample; M, methylated sample. *p≤0.05, **p≤0.001. **B.** Relative mRNA expression in unmethylated versus methylated BCC samples for either *APC* or *SFRP5.* **C.** Microphotographs of selected samples of SHH, APC and RASSF1A. Bar = 200 µm.

To assess whether the methylation status impacts expression of the differential methylated genes, we performed quantitative real-time PCR on primary BCC (n = 6) and normal skin samples (n = 6). Whereas we could not detect *SHH* and *RASSF1A* mRNA in BCC and normal skin samples (data not shown), we did observe that *SFRP5* mRNA expression level was significantly reduced in BCC versus normal skin (P = 0.019). *APC* mRNA expression level was not altered (P = 0.937) in the samples examined (Figure 2A). Nevertheless, BCC with methylated *APC* showed borderline significant reduction mRNA level compared with BCC in which *APC* was unmethylated (P = 0.050). Correlation of mRNA expression and methylation was not observed for *SFRP5* (P = 0.355) (Figure 2B)¸ probably due to the fact that SFRP5 expression levels in all BCC were very low.

To investigate if we could see an effect of DNA methylation on the protein level, we performed immunohistochemical protein staining for SHH, APC and RASSF1A in a random selection of nodular BCC for which the methylation status had also been determined. After extensive testing, we found that commercially available antibodies for SFRP5 were not suitable for immunohistochemistry. Using the hair follicle as internal control (set at 100% positivity), the expression of SHH, APC and RASSF1A in tumor cells was significantly lower (all p-values <0.001) (Figure 2A). The intra-class correlation coefficients (ICC) for the scores of all stainings were high, with 0.87 for SHH, 0.8 for APC and 0.83 for RASSF1A. However, expression levels of methylated samples were not further reduced when compared with unmethylated samples (Figure 2C). Lastly, for SFRP5 we considered the immunohistochemical data available at the ProteinAtlas website by way of independent comparison with our data. For SFRP5, ProteinAtlas has data for one antibody (HPA019840) in six BCC and six squamous cell carcinomas, showing strong cytoplasmic and membranous antibody staining and with strong intensity in >75% of the tumor cells as well as the overlying skin, which is in contrast with the low mRNA expression levels we found (Data S1). We think that the ProteinAtlas samples are affected by overstaining. As a result, it is not possible to conclude from these data whether or not sFRP5 expression in BCC is lower than in unaffected skin, as it is in our series. Furthermore, heterogeneity among immunohistochemical stainings is a frequent problem interfering with any assessment and can be due to several factors including the antibody of choice. To settle this matter, more comprehensive SFRP5 expression analyses in a larger series of BCC, using both immunohistochemistry and quantitative RT-PCR, would be desirable.

### Low Levels of Nuclear ß-catenin Coincide with High Levels of E-cadherin in BCC

**Figure 3:**
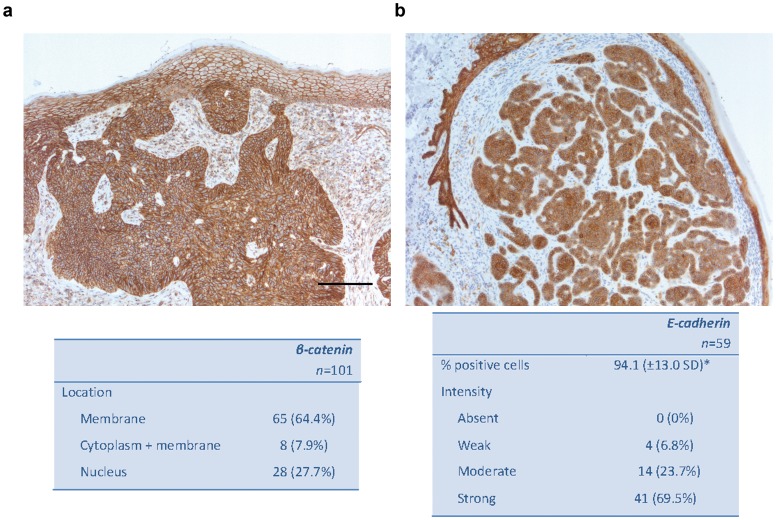
Low levels of nuclear ß-catenin coincide with high levels of E-cadherin in BCC. **A.** Microphotographs of selected sample of ß-catenin, showing nuclear staining only at the periphery or the tumor. Bar = 200 µm. **B.** Microphotographs of selected sample of E-cadherin showing lowered expression of the tumor compared with the normal epidermis.

As our results suggest epigenetic involvement of the WNT pathway in BCC, we subsequently analyzed the expression of β-catenin as readout for WNT pathway activity. β-catenin expression was localized at cell membranes of normal epidermis and within the cytoplasm and nuclei of hair follicles, consistent with the known activity of canonical WNT signaling in this structure [18]. In the 101 BCC we examined, β-catenin was present only at cell membranes in 65 (64.4%) tumors, and localized at both the cell membrane and in the cytoplasm in 8 (7.9%) cases. Nuclear staining was predominantly located at the tumor periphery and only seen in 28 (27.7%) of the BCC samples without preference for one of the subtypes (Figure 3A). No correlation could be detected between the methylation status of *APC* or *SFRP5* and the presence or localization of β-catenin. As it is known that the E-cadherin/β-catenin complex is important in both cell adhesion and canonical WNT signaling [46], we additionally analyzed the expression of E-cadherin, which is normally expressed throughout all layers of the epidermis, including hair follicles [47]. Expression of E-cadherin in 59 BCC was significantly lower compared with the normal epidermis (*P* = 0.001), but was rated high (94.1%) in the tumor cells. Likewise, intensity of staining was rated as strong (69.5%) in the BCC (Figure 3B). These findings were independent of tumor subtype and in agreement with the generally non-metastatic behavior of BCC [3]. The ICC for E-cadherin (0.91) again was high.

## Discussion

To the best of our knowledge, this is the largest study to date addressing epigenetic changes in the most common human malignancy, BCC. We provide data which suggest that the SHH and WNT pathways may be epigenetically involved in BCC pathobiology, since both networks are affected by aberrant promoter methylation of *SHH*, *APC* and *SFRP5*. Aberrant methylation and associated gene silencing of *APC* and *SFRP5* may contribute to the pathogenesis of BCC by impairing negative regulation of WNT pathway activity. In addition, we detected differential methylation of *RASSF1A,* a well-known tumor suppressor modulating a broad range of cellular functions that are essential for normal cell growth. RASSF1A expression is lost in high frequency by promoter methylation in a wide variety of human tumors, in fact, it is one of the best-characterized tumor suppressor genes in UV-induced squamous cell carcinoma of the skin [48], [49] and methylation of its promoter had been previously demonstrated in BCC [28].

Epigenetic alterations in BCC have previously been reported, but in limited sample series [26]–[30] and without providing further experimental evidence for relevance of the epigenetic changes for BCC carcinogenesis. Sathyanarayana et al. investigated promoter methylation of 12 genes in a series of 68 BCC samples and 58 non-malignant lesions (skin tags) and showed that laminin gamma 2 (*LAMC2),* cadherin 1 (*CDH1)* and *RASSF1A* were significantly more often methylated in BCC. These findings are in agreement with our results on *RASSF1A* methylation, even though we found higher methylation frequencies in both BCC and normal skin. A possible explanation for this is that we performed nested MSP, which is a very sensitive technique for methylation analysis on FFPE tissue, while Sathyanarayana et al. performed direct MSP, which is less sensitive. In addition, they investigated the effect of sun exposure on promoter methylation in skin tags and cancer, and found similar methylation frequencies for the genes examined. From this, it was concluded that promoter hypermethylation in general was more likely related to sun exposure rather than being tumor-specific. In our study, we could not detect comparable methylation patterns in sun-exposed skin and BCC for *SHH APC, SFRP5* and *RASSF1A*. Moreover, normal SE and SP skin samples showed similar methylation patterns, suggesting that the methylation we observed is tumor-specific. It is of interest that methylation patterns in sun-exposed skin did not resemble those in BCC, as sun exposure is the main risk factor in the development of BCC [50]. Thus, our findings do not support a contribution of UVB exposure to the observed promoter hypermethylation of *SHH*, *APC*, *SFRP5* and *RASSF1A*. The intriguing observation that nBCC and iBCC harbored more frequent methylation for these four genes compared with sBCC might indicate that DNA methylation contributes to the development or evolution of BCC. It is tempting to speculate that increased silencing of putative tumor suppressors correlates with increasingly invasive behavior, a notion that is supported by our finding of differential *SFRP5* methylation in iBCC versus nBCC and sBCC. The increased *SHH* methylation is probably an epiphenomenon, as SHH is no longer required for SMOH activity in the majority of BCC. As an alternative explanation, *SHH* silencing might contribute through as yet unidentified pathways to tumorigenesis.

To confirm functionally relevant methylation, i.e., transcriptional silencing of affected genes, we performed gene expression analyses with RT-PCR on FF and immunohistochemical staining on FFPE samples with verified methylation status. Our mRNA expression analysis of *APC* and *SFRP5* in patient and control samples confirmed a down regulation of these genes in samples harboring methylation. By immunohistochemistry, expression of *SHH*, *APC* and *RASSF1A* (all p-values <0.001) was lowered in BCC as compared to normal skin. We did not detect a direct correlation between methylation status and expression levels as visualized by immunohistochemistry. We think that this observation can be explained by the two-way detection method in immunohistochemical staining intensifying the original signal. Also, mRNA levels do not always correlate directly with protein levels [51].

These data together support our hypothesis of epigenetic involvement of the SHH and WNT pathways in BCC pathogenesis. Promoter hypermethylation-mediated silencing of negative regulators of WNT signaling is consistent with activity of this pathway, however available literature concerning activity of the WNT pathway in BCC is ambiguous. Therefore we assessed activation of canonical WNT signaling by determining β-catenin intracellular localization. β-catenin, a key WNT effector, is a membrane-bound protein which accumulates in the cytoplasm and subsequently translocates to the nucleus when activated [52]. In BCC, the literature concerning immunohistochemical analysis of β-catenin is ambiguous [19], [53]–[55]. El-Bahrawy et al. [53] showed nuclear staining in 55% (*n = *56) of BCC, mostly at the periphery of the tumors, while only 23% of the 86 BCC examined by Saldanha et al. [54] had β-catenin positive nuclei. No correlation between β-catenin localization and histological subtype was seen in either study. Furthermore, in a paper recently published by Pourreyron et al. [19], nuclear β-catenin was absent in all examined BCC (n = 7) [19]. In our series, we demonstrated nuclear β-catenin in 28 of the 101 (27.7%) BCC examined, which is consistent with previous reports. It would be of interest in this context to examine Axin 2 expression levels in BCC as an additional marker for canonical WNT activity [19]. We reasoned that the lack of β-catenin nuclear localization might be due to E-cadherin co-expression, as high levels of E-cadherin can prevent nuclear translocation of β-catenin [46], [56]. Indeed our data show high levels of E-cadherin expression in BCC, with 94.1% of the cells being positive, although significantly lowered when compared to adjacent normal epidermis. Thus, the absence of nuclear β-catenin in many cases may be due to high E-cadherin levels, which would also be consistent with the general inability of BCC to metastasize [50].

It is of considerable interest that the epigenetic changes we found parallel the genetic changes driving BCC growth, in particular the silencing of known WNT pathway inhibitors. These observations support a biological relevance of gene silencing by promoter hypermethylation in BCC. Moreover, they are consistent with a model in which epigenetic changes help to drive BCC tumor growth through deregulation of the WNT pathway, upon initiation of growth by mutations affecting SHH signaling. Previous work has demonstrated that BCC growth requires WNT signaling [18]–[19]. WNT pathway effectors are among the target genes of SHH, providing a rationale for this positive feedback mechanism. Our findings uncover a new level of regulation; inactivation of WNT inhibitors may be equally important as increased activity of WNT effectors.

In conclusion, we provide evidence that promoter hypermethylation-mediated silencing of known and putative TSGs is present in BCC. The extent to which these epigenetic changes actively contribute to BCC development will be more fully charted in order to determine whether DNA demethylation could be a viable strategy for BCC treatment.

## Supporting Information

Figure S1 **Methylation analysis in three BCC subtypes.** Illustration of ten candidate genes in superfical, nodular and invasive BCC (respectively sBCC, nBCC, iBCC). P-values represent the difference between percentage methylation in the various subtypes.
(DOCX)

Table S1 **Primer sequences and PCR conditions.** Overview of all used primer sequences and PCR conditions.
(DOCX)

Table S2 **Antibody characteristics.** Overview of the used antibodies for all immunohistochemical analysis performed.
(DOCX)

Data S1 **SFRP5 expression in squamous cell carcinoma (SCC) and basal cell carcinoma (BCC) by immunohistochemistry.** Overview of the considerations concerning the data available at ProteinAtlas for SFRP5.
(DOCX)
