# Supplementary figures and images for: Epigenetic Changes in Basal Cell Carcinoma Affect SHH and WNT Signaling Components
Source: PLoS One. 2012 Dec 17;7(12):e51710. doi: 10.1371/journal.pone.0051710 (PMC3524166; doi:10.1371/journal.pone.0051710)

**Figure S1. Methylation analysis in three BCC subtypes.**


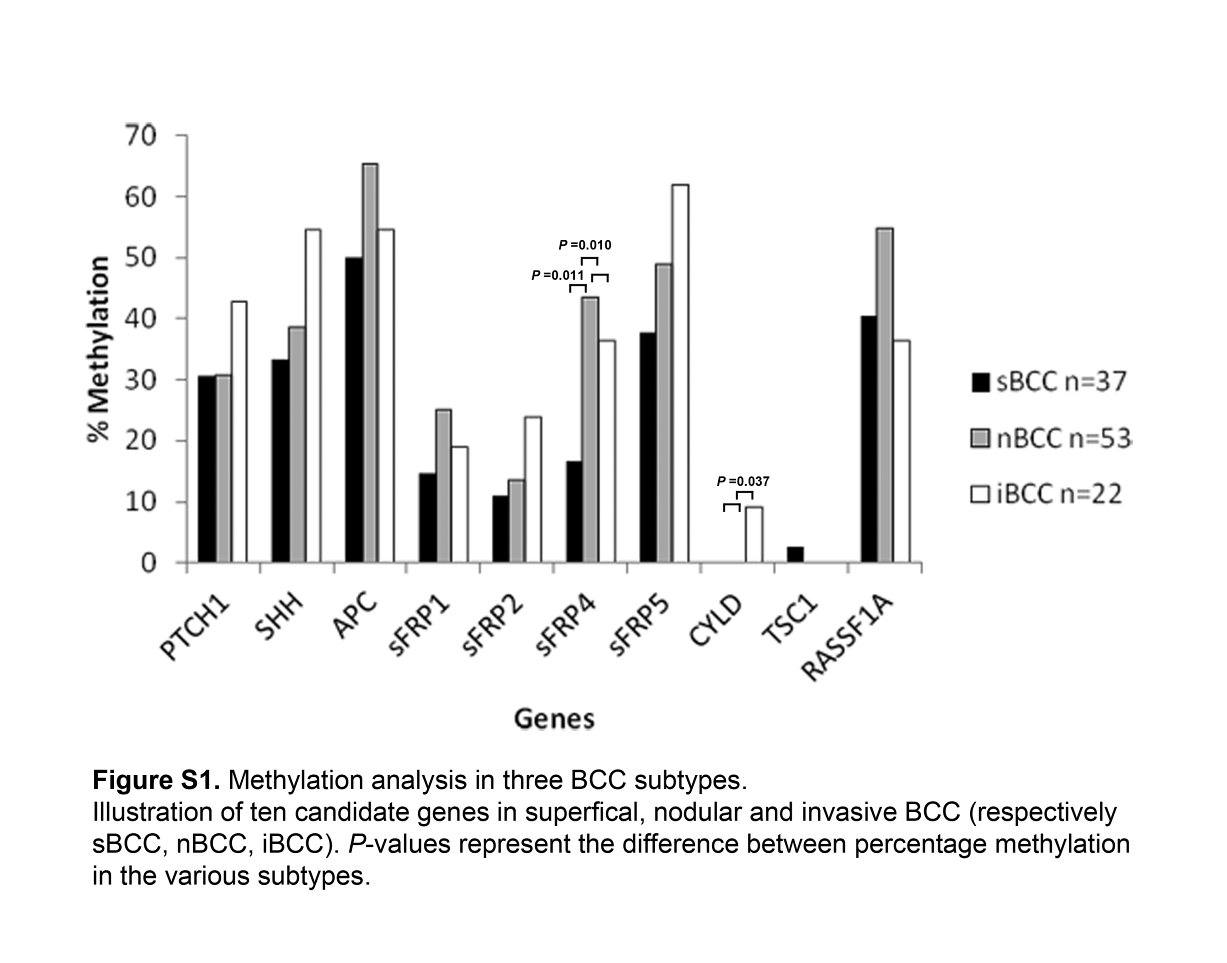

Supplement: Figure S1 — Methylation analysis in three BCC subtypes. Illustration of ten candidate genes in superfical, nodular and invasive BCC (respectively sBCC, nBCC, iBCC). P-values represent the difference between percentage methylation in the various subtypes. (DOCX) [file pone.0051710.s001.docx]
