# Supplementary material for: Epigenetic Changes in Basal Cell Carcinoma Affect SHH and WNT Signaling Components
Source: PLoS One. 2012 Dec 17;7(12):e51710. doi: 10.1371/journal.pone.0051710 (PMC3524166; doi:10.1371/journal.pone.0051710)
Supplement: Table S1 — Primer sequences and PCR conditions. Overview of all used primer sequences and PCR conditions. (DOCX) [file pone.0051710.s002.docx]

**Table S1. Primer sequences and PCR conditions***

| ***Gene*** | ***Primer*** | ***Sense primer***  ***(5’🡪 3’)*** | ***Antisense primer***  ***(3’🡪 5’)*** | ***Annealing temp (ºC)*** | ***No. of PCR cycles*** |
| --- | --- | --- | --- | --- | --- |
| PTCH1 | Flank | GAAGGYGTAGGGTTTGATTTTT | AAAACRCTACTAACCRCAAACTACTC | 56 | 35 |
|  | U | GGTTTGATTTTTTGGTAGTGGTTGT | CTACTAACCACAAACTACTCAAACTCA | 66 | 30 |
|  | M | GATTTTTCGGTAGCGGTCGC | ACCGCAAACTACTCGAACTCG | 66 | 30 |
|  | | | | | |
| SHH | Flank | GTGGGGAGYGGTGGAGAGTTTTT | TCTCTTACRCTTTCCCTTCCTC | 56 | 35 |
|  | U | GTGGAGAGTTTTTTGTAGTTGTGGT | TTCCCTTCCTCACTCCAACTCA | 68 | 25 |
|  | M | AGAGTTTTTCGTAGTCGCGGC | CCTTCCTCGCTCCGACTCG | 68 | 25 |
|  | B | TCRCCCATAAAACTAATAACTTCC | GTGGGGAGYGGTGGAGAGTTT | 64 | 40 |
|  | qRT-PCR | GCCAGCGGAAGGTATGAAGG | TGAGTGGTGGCCATCTTCGT | 60 | 40 |
|  | | | | | |
|  | Flank | TGGGYGGGGTTTTGTGTTTTATT | TACRCCCACACCCAACCAATC | 56 | 35 |
| APC | U | GTGTTTTATTGTGGAGTGTGGGTT | CCAATCAACAAACTCCCAACAA | 60 | 25 |
|  | M | TAT TGC GGA GTG CGG GTC | TCG ACG AAC TCC CGA CGA | 60 | 25 |
|  | qRT-PCR | ATGGAAGCCGGGAAGGATC | TCAGCAAGAAGCAATGACCTCTC | 60 | 40 |
|  | | | | | |
|  | Flank | TTTAGTTTTGTAGTTTTYGG GTTAG | CCCCRACCAATAACRACCCTC | 56 | 35 |
| SFRP1 | U | GTTTTGTAGTTTTTGGAGTTAGTGTTGTGT | CTCAACCTACAATCAAAAACAACA CAAACA | 66 | 25 |
|  | M | TGTAGTTTTCGGAGTTAGTGTCGCGC | CCTACGATCGAAAACGACGCGAACG | 66 | 25 |
|  | | | | | |
|  | Flank | TTTGTTTTTTYGGGTYGGAGTTTTT | TTATCCCRAACCCRCTCTCTT | 56 | 35 |
| SFRP2 | U | TTTTGGGTTGGAGTTTTTTGGAGTTGTGT | AACCCACTCTCTTCACTAAATACAACTCA | 66 | 30 |
|  | M | GGGTCGGAGTTTTTCGGAGTTGCGC | CCGCTCTCTTCGCTAAATACGACTCG | 66 | 30 |
|  | | | | | |
|  | Flank | TTGYGGTTAGAGGGGGTGATG | CTATTTATCCCRACACCTCCCC | 56 | 35 |
| SFRP4 | U | GGGGGTGATGTTATTGTTTTTGTATTGAT | CACCTCCCCTAACATAAACTCAAAACA | 66 | 30 |
|  | M | GGGTGATGTTATCGTTTTTGTATCGAC | CCTCCCCTAACGTAAACTCGAAACG | 66 | 30 |
|  | | | | | |
|  | Flank | GGGAGAGGGGYGTAAGATTTG | CCTCCCACCTCRAAACTCCAA | 56 | 35 |
| SFRP5 | U | GTAAGATTTGGTGTTGGGTGGGATGTTT | AAAACTCCAACCCAAACCTCACCATACA | 66 | 25 |
|  | M | AAGATTTGGCGTTGGGCGGGACGTTC | ACTCCAACCCGAACCTCGCCGTACG | 66 | 25 |
|  | qRT-PCR | ACCGCTGGGACAAGAAGAATA | GCCCCGTAGAAGAAAGGGTA | 60 | 40 |
|  | | | | | |
|  | Flank | GGTTTGTTATAGGGAGGTTTAATTA | CRCACAAAAAAAAAAAAAAAAAC | 56 | 35 |
| CYLD | U | GGTTTGTTTAGGAGTGTAGTTTGG TTTAT | AAAACAAAAACAAAAAAAACAACC A | 66 | 35 |
|  | M | CGTTTAGGAGCGTAGTTCGGTTTAC | ACGAAAACGAAAAAAACAACCG | 66 | 35 |
|  |  |  |  |  |  |
|  | Flank | TYGTAGTTTTTTTTYGGATTTTTT | TCCTTCTCRAAAAAAAAAATAAAC | 56 | 35 |
| TSC1 | U | TTTTGGATTTTTTTGTTTGGTTTT | AAAAAAAAAATAAACAACCACACT CA | 62 | 35 |
|  | M | CGGATTTTTTCGTTCGGTTTC | AAATAAACGACCGCGCTCG | 62 | 35 |
|  | | | | | |
|  | Flank | GTTTAGTTTGGATTTTGGGGGAG | CCCRCAACTCAATAAACTCAAACTC | 56 | 35 |
| RASSF1A | U | GGGGTTTGTTTTGTGGTTTTGTTT | AACATAACCCAATTAAACCCATACTTCA | 64 | 30 |
|  | M | GGGTTCGTTTTGTGGTTTCGTTC | TAACCCGATTAAACCCGTACTTCG | 64 | 30 |
|  | qRT-PCR | GACCTCTGTGGCGACTTCATCT | CGGTAGTGGCAGGTGAACTTG | 60 | 40 |
| CYPA | qRT-PCR | CTCGAATAAGTTTGACTTGTGTTT | CTAGGCATGGGAGGGAACA | 60 | 40 |

PCR, polymerase chain reaction; B, Bisulfite genomic sequencing; U, primers matching originally unmethylated DNA sequence; M, primers matching originally methylated DNA sequence.
