# Supplementary material for: Epigenetic Changes in Basal Cell Carcinoma Affect SHH and WNT Signaling Components
Source: PLoS One. 2012 Dec 17;7(12):e51710. doi: 10.1371/journal.pone.0051710 (PMC3524166; doi:10.1371/journal.pone.0051710)
Supplement: Table S2 — Antibody characteristics. Overview of the used antibodies for all immunohistochemical analysis performed. (DOCX) [file pone.0051710.s003.docx]

**Table S2. Antibody characteristics**

| *Antibody* | ***Source*** | ***Order number*** | ***Positive control tissue*** | ***Dilution*** |
| --- | --- | --- | --- | --- |
| APC | Sigma-Aldrich | HPA013349 | Colon | 1:300 |
| SHH | Abcam | AB53281 | Kidney | 1:50 |
| Rassf1A | Origene | TA502449 | Melanoma | 1:50 |
| β-catenin | Zymed | 18-0226 | Internal control | 1:1000 |
|  |  |  | (hair follicle) |  |
| E-cadherin | Dako | IR059 | Colon | RTU |
