# Supplementary material for: Epigenetic Changes in Basal Cell Carcinoma Affect SHH and WNT Signaling Components
Source: PLoS One. 2012 Dec 17;7(12):e51710. doi: 10.1371/journal.pone.0051710 (PMC3524166; doi:10.1371/journal.pone.0051710)
Supplement: Data S1 — SFRP5 expression in squamous cell carcinoma (SCC) and basal cell carcinoma (BCC) by immunohistochemistry. Overview of the considerations concerning the data available at ProteinAtlas for SFRP5. (DOCX) [file pone.0051710.s004.docx]

**Data S1. SFRP5 expression in squamous cell carcinoma (SCC) and basal cell carcinoma (BCC) by immunohistochemistry**

*Consideration data available at ProteinAtlas online database*

( <http://www.proteinatlas.org/ENSG00000120057/antibody>)

Immunohistochemistry, immunofluorescence, western blot and protein array data on this site are obtained using one antibody: HPA019840 (Sigma Aldrich, St. Louis, USA).

*Immunohistochemistry* shows a staining pattern, which is partly consistent with gene/protein characterization data. Although heavily overstained, the expression is restricted to the basal epidermis and BCC cells and shows no nuclear staining, which confirms specificity. All examined SCC (*n=6)* and BCC *(n=6)* show strong cytoplasmic and membranous antibody staining in > 75% of the tumor cells as well as in the overlying skin. We think that these seemingly high expression levels throughout all samples are likely to be an artifact of overstaining, since all cell types show more or less similar staining patterns.


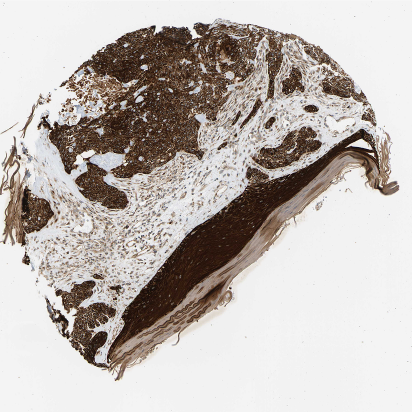

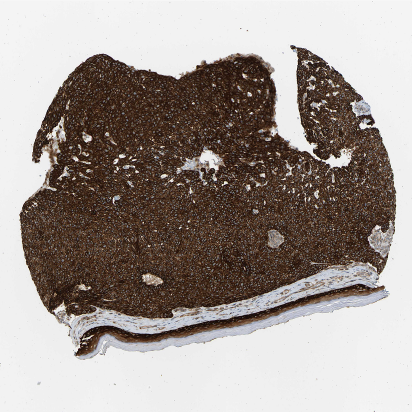


BCC BCC

*Immunofluorescence* of a human cell line, A-431 (epidermoid carcinoma), shows fluorescence in nucleus, cytoplasm and vesicles.

*Western blot* analysis shows a band at the predicted height, corresponding to an MW of 35.6 kDa.

*Protein array* confirms specific binding of the antibody to its antigen.
